# Peri-implant bone changes in immediate and non-immediate root-analog stepped implants—a matched comparative prospective study up to 10 years

**DOI:** 10.1186/s40729-016-0048-0

**Published:** 2016-05-23

**Authors:** German Gomez-Roman, Steffen Launer

**Affiliations:** Department of Prosthodontics, Dental School, University of Tübingen, Osianderstr. 2-8, Tübingen, D-72076 Germanyᅟ

**Keywords:** Immediate implants, Frialit, Peri-implant bone changes, Bone loss, Crestal bone loss

## Abstract

**Background:**

The purpose of this retrospective long-term study was to evaluate the peri-implant bone changes in immediate implants and matched non-immediate implants as a control group using a specific and proven measurement protocol over a 10-year period, because there are no similar studies published.

**Methods:**

One hundred and thirty-three patients received 174 implants (immediate implants (IM) *n* = 87; control group (CG) *n* = 87). The two groups were matched following specific criteria for comparison: implant length, diameter, site of the implant, and patient’s gender. For the evaluation, radiographic images were taken, digitalized, and assessed using the “coronal bone defect (CBD)”.

**Results:**

The differences between the means and medians showed a statistically significant difference at the time of insertion, while to the other control dates, no significant differences could be concluded. The median CBD for the control group was 0 mm at the time of insertion and increased to 1.7 mm after 10 years while the CBD for the IM group was 0.7 mm at the time of insertion and increased to 1.5 mm over the 10 years.

**Conclusions:**

Both surgical protocols lead in our study to similar outcomes regarding the loss of bone around dental implants.

## Background

The success of dental implants has become more and more predictable since Brånemark first observed what he later called osseointegration, in 1960 [1], meaning the direct structural and functional interlocking of the natural bone and titanium implant surfaces. With implantation becoming a predictable treatment for dental restorations, patients also have become more critical towards the esthetic outcome and the longevity of the restorations. At the same time, clinicians seek for insertion techniques which can reduce the number of surgeries needed from tooth removal to the final restoration.

**Fig. 1:**
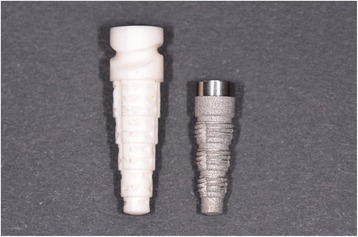
On the *left*, the Tübingen ceramic implant; on the *right*, the Frialit stepped-screw implant. The transgingival part with the cervical groove of the Tübingen implant has been removed in the Frialit implant; this part is now replaced by a mirror-polished transgingival portion of the Frialit abutment; all the intraosseous portion of the implant has now a thread and is shaped like the original Tübingen implant

When in 1975 Professor Schulte and his team at the Eberhard Karls University in Tübingen first introduced the concept of immediate implantation in fresh extraction sockets, it seemed promising at first [2]. However, the use of a full aluminum-oxide ceramic implant (Tübingen implant, Friedrichsfeld, Mannheim, Germany, shown in Fig. 1 on the left side) with small lacunae for bone apposition rather than a screw-shaped profile led to an intolerable rate of early implant failure.

Today, almost 40 years after the introduction of the concept of immediate implantation, there have been a lot of developments regarding insertion technique as well as structural changes of the implants and their surfaces and immediate implantation has become a concept widely accepted and proven successful in many studies [3–5].

This success is often assessed by referring to survival rates. However, a more precise method is the determination of the rate of osseointegration over a long-term period. Many articles, although using radiographic images to assess the bone-level changes, fail to provide the used measurement protocol. An adequate comparison between two or more images can only be achieved by using a constant reference point and reference length as well as a factor to eliminate the distortion factor often present in radiographic images. Further, this is the only way to compare results found by different working groups, or the same group, in different examinations.

The present study was designed as a retrospective long-term study which compares the peri-implant bone situation of immediate implants and non-immediate implants as a control group (matched with specific criteria) using reliable measurement specifications [6–8]. The hypothesis was that both the immediate implantation and the implantation after a healing period using the Frialit 2 implant system lead to similar results.

## Methods

All patients within this study were treated at the Dental School of the Eberhard Karls University in Tübingen between the 22nd of February in 1991 and the 24th of October in 2005. Every patient received at least one Frialit implant. The study protocol of the study was approved by the German Society of Research (Sonderforschungsbereich 175 Implantologie). Informed consent was obtained from all patients. The study evaluates the Frialit implants (Friadent, 68221 Mannheim, Germany) shown in Fig. 1, right side.

Patient selection criteria:

This study includes patients matching the following criteria:

- Patients need to agree to take part in this study as well as being part of a regular recall system.
- Patients must have a need for dental implants, such as edentulous ridge, distal-extension situation, tooth bound gap, or a single tooth replacement. This includes patients with tooth loss due to traumata, excessive internal tooth resorption, endodontic failure, root resorption after replantation, or retained primary teeth (in case of agenesis) as well as patients with tooth loss because of excessive caries or advanced periodontitis.
- Implants that were inserted immediately and a matched control group of implants that were inserted after a healing period of at least 3 months after tooth extraction (non-immediate implants).
- The matching criteria: implant length, diameter, site of the implant, and the patient’s gender.
- Radiographs from either the time of insertion and/or the time patients received their restorations as well as follow-up radiographs had to be available.

Patients were ineligible if one of the following criteria was matched:

- Irradiation of the implant area
- Pathologic changes of the receptor site (cysts, tumors, osteomyelitis, etc.)
- Insufficient bone volume
- Acute periapical pathology (tooth sensitive to percussion)
- Existence of non-treated generalized progressive periodontitis
- Chronic acute systematic disorders (e.g., uncontrolled diabetes, hemorrhagic diatheses, general or auto immunodeficiency)
- Patients refusing to give their consent for the use of their data

One hundred and thirty-three patients receiving 174 implants were selected: 87 immediate implants (IM) and as a matched group 87 implants that were inserted in healed bone (control group (CG)).

**Fig. 2:**
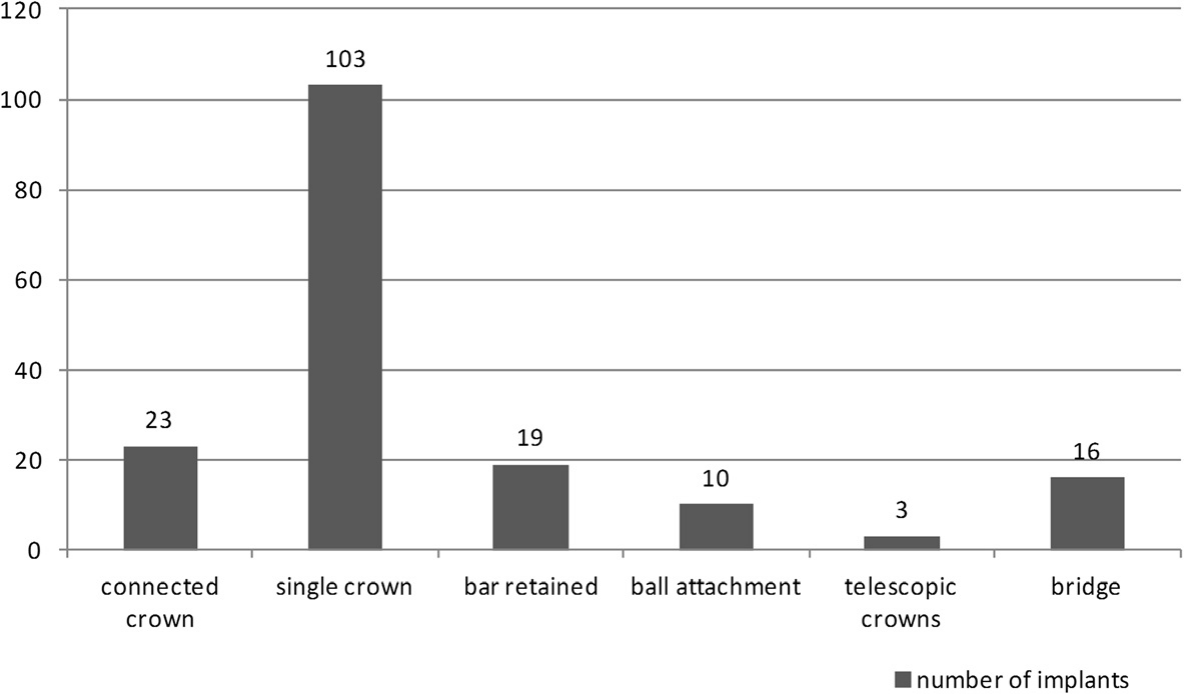
Prosthodontic indications

The mean age of the patients in this study was 42 years, the youngest patient being 15 years old and the oldest 75 years at the time they received their implant. A gender and age distribution of all inserted implants is outlined in Table 1. Figure 2 shows a distribution of the prosthodontic indication. The prosthodontic indication was no matching criteria, and therefore, Fig. 2 shows an overview over all the implants and their prosthodontic indications in this study.

**Table 1:** Distribution of implants according to the gender and age of the patient at the time of implant insertion

	Age (year)			
Gender	15–20	21–40	41–60	61–75
Females (*n*)	14	25	27	14
Males (*n*)	6	35	36	17
Total (*n*)	20	60	63	31
Percentages	11	34	36	18

**Fig. 3:**
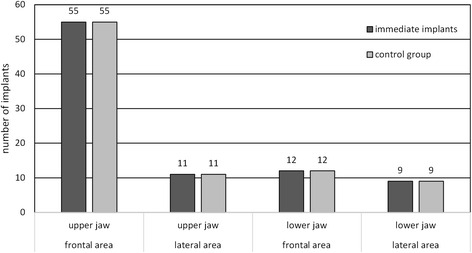
Number of implants in the respective region (anterior region ranging from 13 to 23 and 33 to 43, and posterior region ranging from 18 to 14, 24 to 28, 38 to 34, and 44 to 48)

In total, 133 patients received 174 implants. This number equally consists of immediate and non-immediate implants. For every immediate implant, a control implant from the non-immediate group was needed. For an ideal comparison, this control implant had to match some criteria. In a 2000 published thesis with a great number of implants, it was shown that the crucial criteria for comparing the survival of implants are the diameter and the location of the implant as well as the gender of the patient [9]. Figure 3 gives an overview of the implants and their locations while Table 2 shows the number of implants in regard to the diameters and lengths.

**Table 2:** Number and dimension of the implants

	Length (mm)		
Diameter	10	13	15
3.8 mm	0	4	52
4.5 mm	4	8	54
5.5 mm	0	8	38
6.5 mm	0	0	6

Conventional radiographs were taken right after implant insertion, the day patients received their prosthetic restoration and further on in irregular intervals whenever indicated or necessary for other treatments. A total of 974 radiographic images of implants were assessed in this study, 408 were panoramic radiographs and 566 were intraoral radiographs.

The radiographs where digitalized using a transmitted light scanner (Intelli Scan 1600, QUATOGRAPHIC Technology GmbH, 38112 Braunschweig, Germany) using the Silverfast AI scanprogam (Version 6.4 LaserSoft Imaging AG, 24105 Kiel, Germany) and imported into the Sidexis XG program (Sirona, 64625 Bensheim, Germany). For an ideal result of the scanned images, they were imported with a resolution of 300 dpi and a 16-bit grayscale. The resulting digital images had to be converted from .tiff files to .bmp files before importing them into the Sidexis program.

**Fig. 4:**
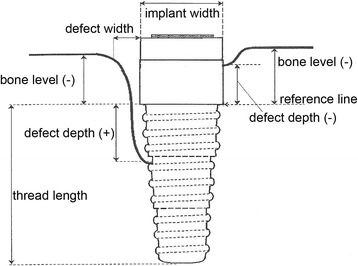
Measurement specifications: outline [6]

**Fig. 5:**
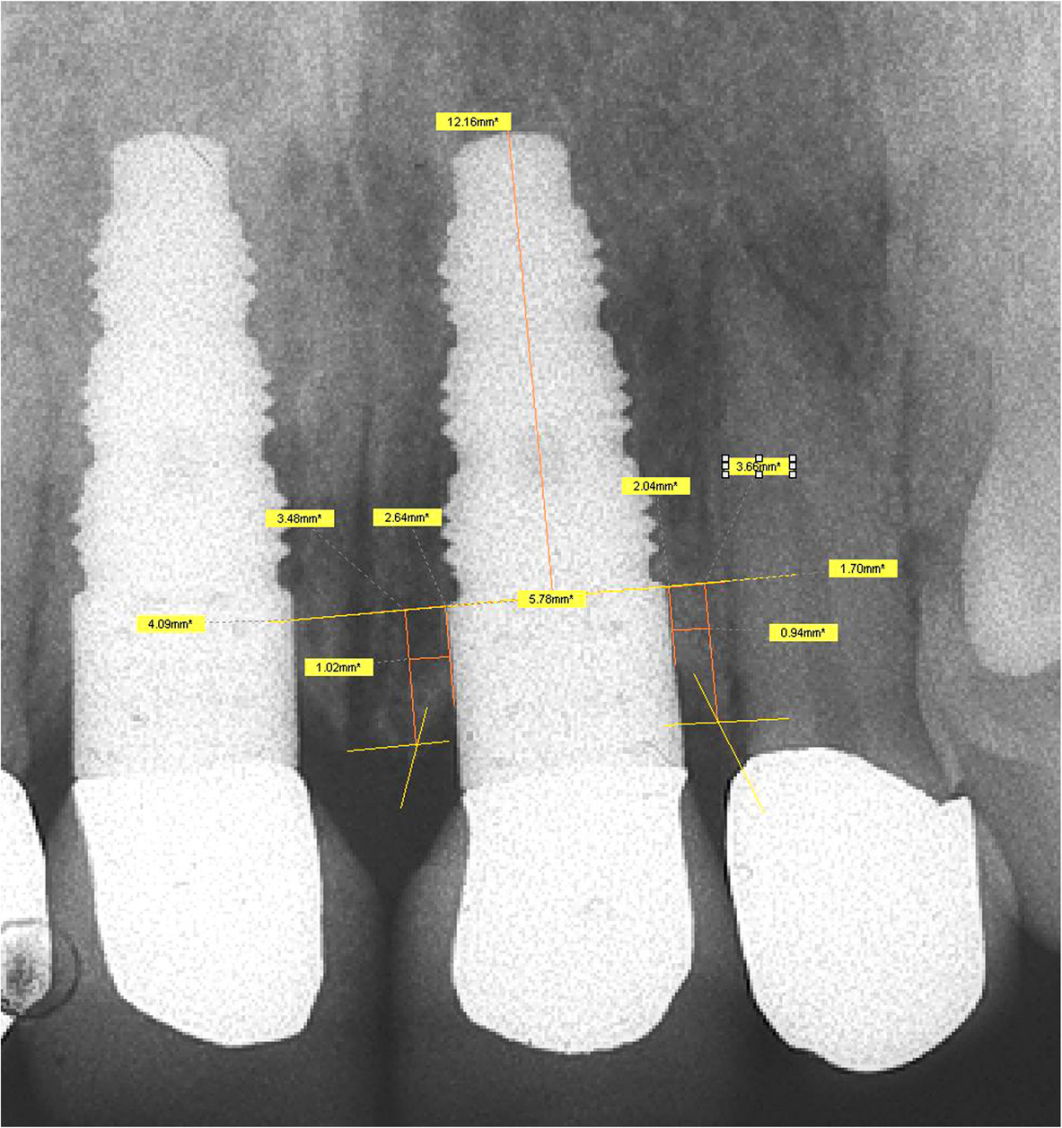
Measurement specifications: clinical realization [6]

The protocol used for measuring the distances in every radiograph was described by the author [6] and is outlined in Figs. 4 and 5. Crucial is the determination of a reliable reference line for every implant type. Rather than measuring only the bone level, the “coronal bone defect,” described by the author in 1995 [6], is assessed, which is the extent to which the part of the implant that is meant for osseointegration failed to be osseointegrated.

All these distances were recorded on special evaluation forms, independent for each radiograph.

The obtained data was transmitted to the SAS JMP program (JMP 9, SAS Institute, Cary, USA) for further processing.

Since radiographic images are known to have a distortion, all distances had to be multiplied with a factor that was individual for each radiograph. Therefore, the length of the most coronal step was needed. When subtracting this number from the implant length provided by the manufacturer, the exact length from the apical point of the implant to the reference line is obtained. Dividing the distance gathered from the radiograph with the absolute distance provides the factor that is needed to convert all distances measured vertically. The factor for the horizontal distances is similarly obtained by using the measured implant diameter and the real implant diameter provided by the manufacturer.

The formula used for calculating the coronal bone defect (CBD) is provided in Table 3. The different values added to the depth of the bone defect (DD) are based on the various lengths of the upper most part of the Frialit.

**Table 3:** Formula for the CBD

Implant diameter and length	Formula for the calculation of the CBD
	(CBD = coronal bone defect)
	(DD = defect depth)
3.8 × 13 mm	CBD = DD + 5 mm
3.8 × 15 mm	CBD = DD + 7 mm
All other diameters	CBD = DD + 3.2 mm

After converting the data from the measured lengths (radiolucency) to the “coronal bone defect” by calculation, the results for the mesial and distal parts were plotted in a chronological sequence. This was carried out to check for outliers and also served as an assessment of plausibility. If an anomaly was found, the radiographs were measured again and the documentation forms were reassessed. Using this technique, errors due to false transmission or measurement errors could be identified and rectified.

After this, the data set was imported into the Excel program (Microsoft Corporation, Redmond, WA 980526399, USA) for further breakdown. For the final examination of the observed values, the mesial and distal CBD was compared. Since there were no larger differences of the values, the mesial and distal CBD were averaged and this was used for further assessment. The gained results were visualized using box plots according to Tukey [10] with a limit for the whiskers being 1.5 times the interquartile range (IQR). Values exceeding these numbers were marked as outliers.

### Statistical methods

The statistical tests used to verify the hypothesis of this paper were the *t* test and the Wilcoxon test.

## Results

For the evaluation of the received data, immediate and non-immediate implants were first examined separately.

**Fig. 6:**
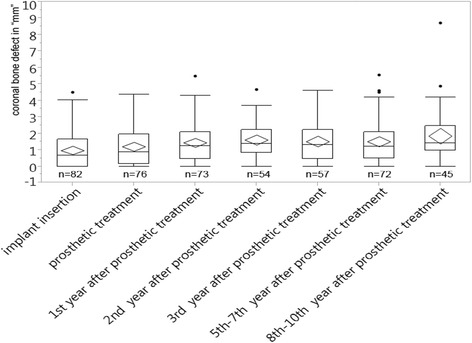
CBD in millimeters plotted over 10 years for the immediate implant group

The immediate implant (IM) group and its CBD plotted against the time, starting at the time of insertion, is shown in Fig. 6. One can see that the data are quite homogeneous.

The arithmetic means of the CBD range from 1.0 to 1.9 mm, most of them lying between 1.1 and 1.5 mm. The lowest mean is found at the time of insertion (1.0 mm) and the highest in the years 8–10 (1.9 mm). At the time of the prosthetic treatment, the mean is at 1.2 mm.

After the time the patients received their prosthodontics, the difference among the means of the CBD was 0.4 mm at its highest (Fig. 6).

For the medians, a similar stable trend could be observed; however, the average values for the medians were found to be a little smaller than the ones for the means, thus indicating asymmetry in the distribution of the data.

The median value for the immediate implants at the time of insertion was 0.7 mm, while at the time when the patients received their restorations, it was found to be 0.9 mm. Medians varied from 0.7 mm at insertion up to 1.4 mm in the second year and the years 8–10 after the restorations were inserted (Fig. 6).

Figure 6 also shows that most of the medians were settled between 0.9 and 1.3 mm. The difference within the medians after the time patients received their prosthodontics was at 0.2 mm at its highest.

The range of the quartiles turned out to be stable. For the upper quartile, values ranging from 1.7 to 2.5 mm were found. Most of them, however, were found to lie within 2–2.2 mm. The lower quartile ranged from 0 to 1 mm. Like for the upper quartile, the majority lay closer together meaning between 0.5 and 1 mm. After the day of the restoration, the values found were a little higher but yet remained stable among each other (Fig. 6).

**Fig. 7:**
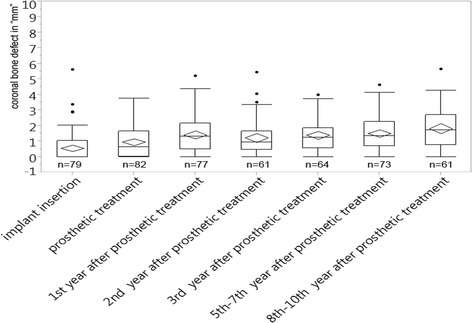
CBD in millimeters plotted over 10 years for the control group

The corresponding group of non-immediate implants was named the control group (CG) and analyzed the same way, and the results are shown in Fig. 7.

Like the immediate implant group, the mean values for the CG mainly stayed stable. At the time of insertion, a value of 0.6 mm was found, which was a little smaller than the value found at the time of the prosthetic treatment (1.0 mm). The maximum value reached for the mean CBD is 1.8 mm (eighth to tenth year). Most of the mean values scattered close around 1.3 mm (Fig. 7).

The median values showed a similar behavior. While after the insertion a median of 0 mm is found, this value increased over the time being at 0.6 mm for the time patients received their restorations and later lay between 0.9 and 1.7 mm (Fig. 7).

**Fig. 8:**
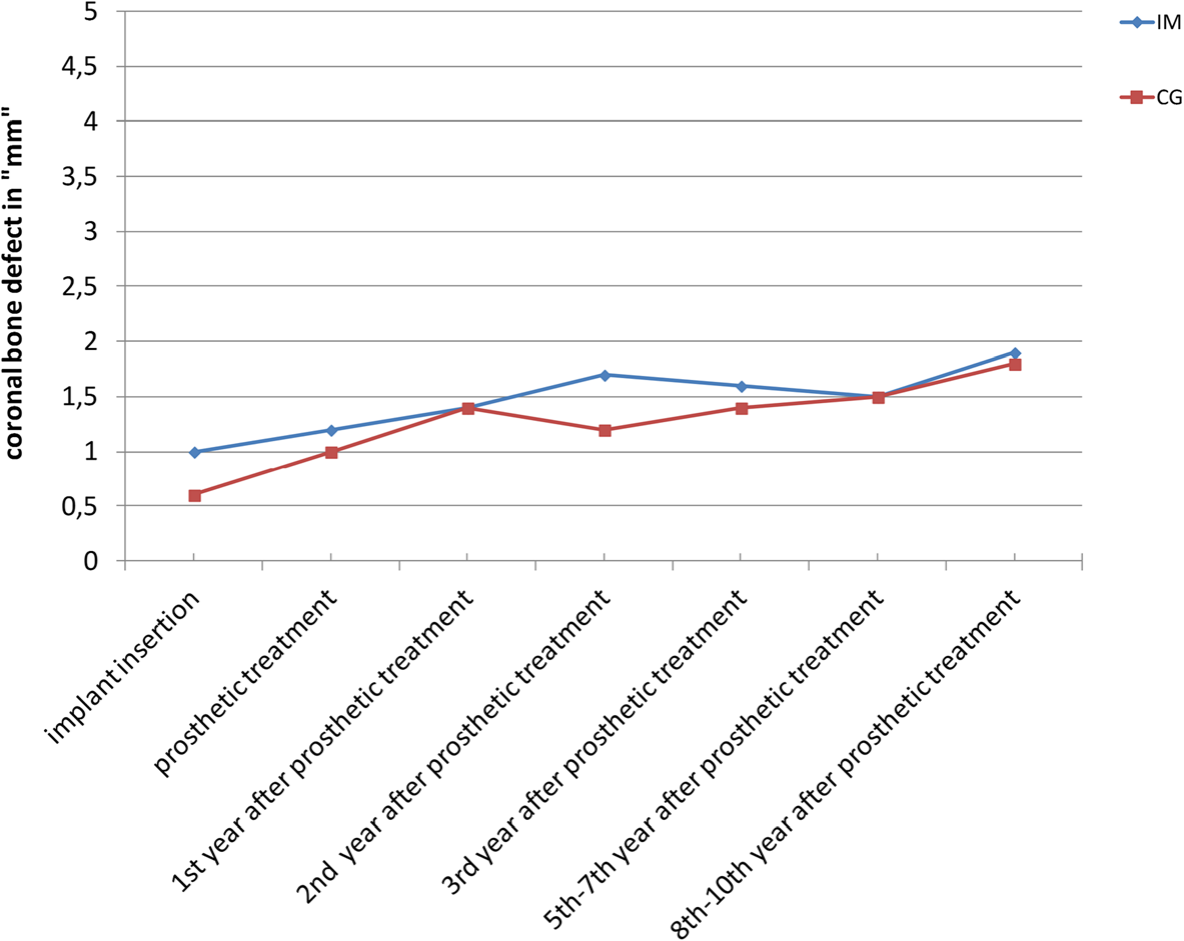
Comparison of the CBD means in the immediate implant and the control groups

**Fig. 9:**
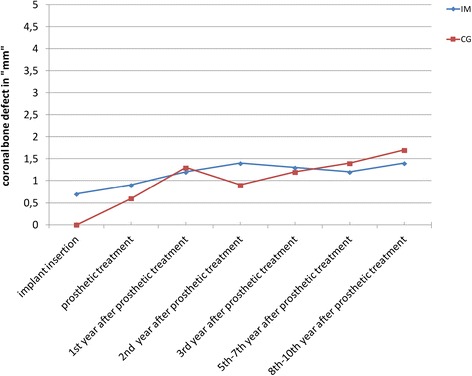
Comparison of the CBD medians in the immediate implant and the control groups

Figures 8 and 9 compare the means and medians of the immediate and non-immediate groups, respectively.

The diagrams show the means and medians of both groups (IM and CG), respectively, in one figure, with the mean values for the IM group being a little higher than those in the CG until the fifth to seventh year after prosthetic treatment (Figs. 8 and 9).

For the medians (Fig. 9), both groups show increasing values until the first year after patients received their definite restorations. Striking is the comparison of the median values as the median values at the time of insertion were significantly higher in the IM group than those in the CG (incongruence between the shape of the alveolus and the implant body).

In the second and third year after the restorations were emplaced, the median CBD values for the CG were found to be a little smaller than for the IM group. And for the last control period, this observation was found to be inverted meaning the median CBD values for the IM group were found to be smaller than the ones for the CG (Fig. 9).

Comparing the medians (Fig. 9) of the IM and CG, a difference of 0.7 mm at the time of insertion is visible. The respective values were 0 mm for the CG and 0.7 mm for the IM group. While at the time the patients received their definite restorations, a difference of only 0.3 mm was recorded (0.9 mm for IM and 0.6 mm for CG).

Over the years after definite restorations were emplaced, the medians for both groups mainly lay between 1 and 1.7 mm, the difference mostly being less than 0.5 mm (Fig. 9).

In summary, the mean and the median values stayed stable over the follow-up years. Both groups showed a slight increase of the mean and median values from the beginning of the study until the eighth to tenth year and mainly ranged from 1 to 1.7 mm.

The upper quartiles of the CG showed a tendency for smaller values up to the third year after restoration, while later, that tendency was no longer detectable. For the lower quartile, there was no significant difference visible (Fig. 7).

Statistical tests, shown in Table 4, were carried out to compare the data sets. A statistically significant difference between the two data sets was found at the time of insertion in all three tests, and the second year after patients had received their restorations, a rejection of the null hypothesis for the Wilcoxon test could be observed.

**Table 4:** Analysis of the statistical tests

IM vs. CG	Insertion	Prosthetic treatment	1st year after prosthetic treatment	2nd year after prosthetic treatment	3rd year after prosthetic treatment	5th–7th year after prosthetic treatment	8th–10th year after prosthetic treatment
Null hypothesis *t* test	Rejected	Accepted	Accepted	Accepted	Accepted	Accepted	Accepted
*P* value	0.01	0.15	0.91	0.06	0.56	0.97	0.93
Null hypothesis Wilcoxon test	Rejected	Accepted	Accepted	Rejected	Accepted	Accepted	Accepted
*P* value	0.01	0.2	0.93	0.02	0.83	0.56	0.7

## Discussion

The primary objective of this study was the assessment of the peri-implant bone situation in immediate implants over a long-term period up to 10 years and to compare it to the situation found in matched non-immediate implants because there are no similar studies published. The hypothesis was that both the immediate implantation and the implantation after a healing period using the Frialit 2 implant system lead to similar results.

The uneven number of patients is due to the inclusion criteria. All patients that could be matched and were eligible for radiographic assessment were selected for evaluation.

This study evaluates implants in the every region of the human jaw. Due to the matching of every implant with an implant following the criteria set by the author, both implant groups remain comparable [9].

For the inclusion to this study, radiographs of the patients’ implants had to be present for either the time of insertion or the day patients received their definite restorations. For the time after prosthetic treatment, radiographs were taken whenever indicated or for other treatments in order to keep the exposure time to radiation as small as possible.

Due to the use of a strict and reliable measurement protocol [6–8] and the direct plotting of the found values for the bone defects, errors could be identified and reduced to a minimum. The anomalies found mainly occurred due to transcription errors while importing the data from the evaluation forms into the SAS JMP program. For outliers that could not be explained that way, the respective radiograph was reassessed and the measurement step was carried out again and the values corrected whenever needed. The different numbers of radiographs at the time of insertion in both groups are explainable by different reasons. Some radiographs even though acceptable for clinical use could not be assessed in this study either because of overlapping effects from structures adjacent to the implant or if the most apical part of the implant itself was not detectable on the radiographs the radiograph could not be assessed. In order to keep the exposure to radiation as low as possible, radiographs that provided the clinically relevant information were not repeated even though they could not be included in this study.

The fact that the used measurement protocol is reliable has been proven in former studies [7, 8].

The use of intraoral radiographs as well as panoramic radiographs is an accepted method for the peri-implant bone evaluation [11–13]. The radiographic distortions present in panoramic imaging are well known; however, according to several studies, panoramic images are suitable to assess the crestal bone situation [14, 15]. According to Zechner et al., rotational panoramic and intraoral rectangular radiographs lead to comparable outcomes [16]. However, this is only possible on the mesial and distal sides. While the possibility to assess the bone in every dimension seems tempting, the higher exposure to radiation needs to be considered. That the mesial and distal defects can be averaged is supported by the work from Cooper et al. in 2010 [17].

Current studies rarely evaluate the peri-implant bone situation using a clearly defined measurement protocol and therefore are often sensitive to measuring errors. Due to using a clearly defined and proven measurement protocol, abnormalities were mainly caused by transcription errors and could be identified in the plotting step. There is no comparable study over such a period of time with two groups that were matched following specific criteria.

Although several studies report a better healing for immediately placed implants and a lower rate of resorption for the surrounding bone compared to delayed immediate implants or implants placed in healed sites [17–22], the results obtained in this study do not support this thesis. The differences found to the respective points of time show comparable results for both groups.

The results of Covani et al. suggest that the process of bone remodeling cannot be influenced by changing the time of implantation but merely by the implant position [19]. These results compare to the results found by Deng et al. [23]. They reported an initial bone resorption of 1.01 mm for immediate implants within the first year, which is similar to the results found in this study.

Studies that evaluate the peri-implant bone situation specifically in the Frialit 2 implant system were, among others, conducted by Krennmair et al. and Ricci et al. Krennmair et al. found a bone resorption of 1.4 ± 1.2 mm over their follow-up period [24], which compares to the results found in this study. Ricci et al. found a higher bone resorption of the crestal bone after a 5-year follow-up, being 2.17 ± 1.6 mm in average [25]. Noteworthy is the high percentage of implants showing a bone loss of more than 3 mm (28.6 %).

One possibility for the outlier values could be a peri-implant defect with less than three walls. These defects tend to have a lower chance of spontaneous healing [5].

When comparing the data sets in our study, there is no deviation from the range of measured tolerance being 0.5 mm for the medians or means among the two groups. The median value for the CBD lies significantly higher for the immediate implants compared to the median value for the control group at the time of insertion. One possible explanation could be the incongruity between the shape of the alveolus and the shape of the implant, thus resulting in a possibility of a contact point, between bone and implant, lying closer to the reference line and therefore showing a higher value for the CBD. The fact that the null hypothesis for the Wilcoxon test was rejected in the second year after prosthodontics could be a result of the assumption of the test. Since the test assumes two groups with distributions different from the normal distribution.

That immediate and non-immediate placements of Frialit-type implants can achieve similar results was reported by Perry and Lenchewski in 2003. Perry and Lenchewski reported on a similar survival rate for Frialit implants placed as immediate implants and in healed sites [26]. However, in his study, there was no radiographic evaluation of the surrounding bone situation.

When Quirynen et al. did their review on how the time difference between extraction or tooth loss and implantation affects the success of the implant, no significant difference could be found [27]. They as well as Ortega-Martínez et al. clearly demand more studies evaluating the bone situation for the future [28]. This question was the motivation for our study.

## Conclusions

The examination and comparison of the peri-implant bone situation in immediate implants and a control group of non-immediate implants that were matched following specific criteria over a long period of time (10 years) has shown statistically significant differences only at the time of insertion and for the Wilcoxon hypothesis in the second year after prosthetic treatment. In our study, immediate implantation leads after an observation period to similar outcomes regarding the loss of bone around dental implants as the conventional placement in healed sites with bone coronal bone defects ranging from 1.3 to 1.7 mm in the follow-up years after prosthetic treatment.

## Abbreviations

CBD: coronal bone defect (part of the implant that has no contact to the bone; a calculated value)
CG: control group
DD: defect depth visible in the radiographs
dpi: dots per inch
IM: immediate implant group
IQR: interquartile range
